# K^+^ vs. Na^+^ Effects on the Self-Assembly of Guanosine 5′-Monophosphate: A Solution SAXS Structural Study

**DOI:** 10.3390/nano10040629

**Published:** 2020-03-28

**Authors:** Enrico Junior Baldassarri, Maria Grazia Ortore, Francesco Spinozzi, Adam Round, Claudio Ferrero, Paolo Mariani

**Affiliations:** 1 Marche Structural Biology Center, Department of Life and Environmental Sciences, Università Politecnica delle Marche, 60131 Ancona, Italy; enricojrbaldassarri@gmail.com; 2 Department of Life and Environmental Sciences, Università Politecnica delle Marche, 60131 Ancona, Italy; m.g.ortore@univpm.it (M.G.O.); f.spinozzi@univpm.it (F.S.); 3 European XFEL, SPB/SFX Instrument, 22869 Schenefeld, Germany; adam.round@xfel.eu; 4 European Synchrotron Radiation Facility—E.S.R.F., 38043 Grenoble, France; ferrero@esrf.fr

**Keywords:** G-quadruplex, nanowire, telomeric assembling, metal cation effects

## Abstract

The hierarchical process of guanosine (G) self-assembly, leading in aqueous solution and in the presence of metal cations to the formation of G-quadruplexes, represents an intriguing topic both for the biological correlation with telomerase activity and for the nano-technological applications, as demonstrated by the current measured in a quadruplex wire 100 nm long. Similar to G-rich DNA sequences and G-oligonucleotides, the guanosine 5′-monophosphate (GMP) self-aggregates in water to form quadruplexes. However, due to the absence of a covalent axial backbone, this system can be very useful to understand the chemical-physical conditions that govern the guanosine supramolecular aggregation. We have then investigated by in-solution Synchrotron Small Angle X-ray Scattering technique the role of different cations in promoting the quadruplex formation as a function of concentration and temperature. Results show how potassium, with its peculiar biological traits, favours the G-quadruplex elongation process in respect to other cations (Na${}^{+}$, but also NH${}_{4}^{+}$ and Li${}^{+}$), determining the longest particles in solution. Moreover, the formation and the elongation of G-quadruplexes have been demonstrated to be controlled by both GMP concentration and excess cation content, even if they specifically contribute to these processes in different ways. The occurrence of condensed liquid crystalline phases was also detected, proving that excess cations play also unspecific effects on the effective charges on the G-quadruplex surface.

## 1. Introduction

**Figure 1:**
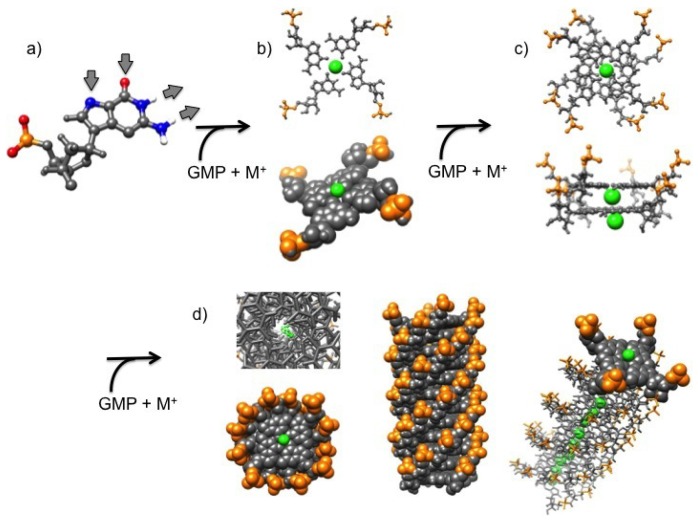
Guanosine self-assembly process in absence of axial backbone. Green: counterions (also indicated as M${}^{+}$); orange: phosphate groups. (**a**) the guanosine 5′-monophosphate, GMP. The donor and acceptor groups are indicated by the arrows; (**b**) the G-quartet (ball-and-stick and van der Waals models); (**c**) the G-octamer (ball-and-stick model), top and lateral views; (**d**) the G-quadruplex (ball-and-stick and van der Waals models), top and lateral views.

Guanine (G) has gained considerable interest in the last years because of the four DNA bases is the only one able to give rise to supramolecular aggregation phenomena in solution, leading to complex structures ranging from G-quartets to G-quadruplexes and G-ribbons [1,2,3,4,5,6,7]. G-quartets and G-quadruplexes form by self-assembly in water solution: G-quartets are planar rings made by four guanine molecules bound to one another through extended hydrogen bonds (according to the so-called Hoogsteen’s scheme) (Figure 1a,b), while G-quadruplexes are four-stranded helical structures made by the out-of-register stacking of planar G-quartets coordinated by monovalent cations (Figure 1c,d). Length and stability of G-quadruplexes have been observed to be related to water concentration, temperature and presence of metal ions, as they fill the G-quartet inner cavities and enhance base-stacking forces [1,2,8,9].

Several low-molecular weight guanine derivatives (as guanosine 5′-monophosphate, GMP [10]) have been detected to share this behaviour, but even guanosine-rich single stranded sequences of DNA can adopt similar structures in solution. Quadruplexes forming sequences were indeed identified in eukaryotic telomeres and in a number of non-telomeric genomic DNAs. In particular, quadruplexes can be formed via intramolecular assembly or by association of two or four filaments, according to different geometries [11]: even if such guanosine-rich sequences have focused the attention of the scientific community due to the potential applications in the fields of medicine and chemistry, the role of G-quadruplexes *in vivo* is still not satisfactory resolved, possible because most of the published studies refer to synthetic oligomers, restriction fragments, or recombinant plasmids in cell-free systems. However, one main and common feature of G-quadruplexes has to be underlined: they can be a very good target for a new class of anti-cancer drugs, as they have been demonstrated to be correlated with the telomerase activity [12,13]. Besides this, G-quadruplexes have drawn attention in nanotechnology due to other peculiar properties, too. At one side, stable and transparent supramolecular hydrogels, with water volume fractions larger than 0.95, have been observed to form in binary mixtures of GMP and guanosine [14,15]. At the other side, G-quadruplexes have been successfully tested as molecular nanowires for nanoelectronics [16]. Compared to double-stranded DNA, the tetrameric stacking is expected to provide better conditions for the π-overlap and better stability under various external conditions, while the characteristic low ionization potential of guanine increases the probability of charge transport through the nanowires [17,18].

An important point concerning guanosine supramolecular association is related to the G-quartet stability. Actually, the formation of G-quadruplexes needs the presence of monovalent cations that, by coordination with the oxygen atoms in the inner cavity between two faced G-quartets, promotes the stacking of the tetrads [19]. The cations must have two special features: a positive monovalent charge and an appropriate size to fit into the inner cavity inside the G-quadruplex. Not all the cations are effectively able to fit these conditions, hence they were evaluated and classified according to their stabilizing ability [20]:

$$\begin{array}{c} K^{+}>{\mathrm{Rb}}^{+}>{\mathrm{Na}}^{+}>{\mathrm{Cs}}^{+}={\mathrm{Li}}^{+}. \end{array}$$

Nevertheless, there is a common concept that Li${}^{+}$ destabilizes the stacking, or plays a neutral role. In addition to the alkali metal ions, the non-metallic monovalent cation NH${}_{4}^{+}$ has been shown to stabilise G-quartets to an extent similar to that observed for Na${}^{+}$ [21]. The ionic radius is considered one of the major factors controlling the stabilising ability of cations for quadruplex formation. Na${}^{+}$ (ionic radius 0.95 Å) is small enough to be coordinated with the four O${}_{6}$ atoms within the plane of a G-quartet, while ions such as K${}^{+}$ and NH${}_{4}^{+}$ (ionic radii 1.33 and 1.48 Å, respectively) are too large to be coordinated in the plane of a G-quartet. However, their positioning between two stacked G-quartets and coordination with eight O${}_{6}$ atoms from the two G-quartets have been demonstrated [20].

K${}^{+}$ and Na${}^{+}$ play a key role because they are the major intracellular and extracellular ions, respectively. A possible idea is that these two cations may regulate a switch process between two DNA forms in the cells: linear and G-quadruplexes, as described by Sen and Gilbert [22].

For all these reasons, the interest around G-quadruplexes has been gradually increasing in the last years [17,18,23], with special emphasis to those obtained by low-molecular weight derivatives, as GMP. Unlike G-rich DNA sequences and G-oligonucleotides, in these systems G-quartets are stacked on one another at a constant distance of about 3.4 Å even if they are not covalently linked. This fact indicates that stacking interactions and counterion coordination in the tetramer cavity are providing a suitable force for building stable and long structures. However, due to the absence of the covalent axial backbone, a number of specific properties appears, such as a strong dependence of the quadruplex length on concentration and temperature [10], and a deep relationship between stacking stability and amount and kind of counter ions present in solution. It should be then clear that GMP can be considered a very suitable model to extract information on the guanosine self-assembly process and on G-quadruplex structure, elongation and stability.

The study of such a system has always been particularly complex, owing to three main factors: the very intricate phenomenon of hierarchical aggregation, the large number of parameters controlling the G-quadruplex nucleation (GMP concentration, temperature, counterions effect) and the concomitant elongation/fragmentation processes [10]. In this paper, we then analysed separately the effect of the different parameters in GMP self-assembly by in-solution Small Angle X-ray Scattering (SAXS). SAXS experiments were performed in different conditions, e.g., as a function of GMP concentration, type and content of counter ions in solution and temperature, and data were analysed by taking into account that G-quadruplexes share a number of specific structural characteristics (e.g., diameter and electron density distribution) and that in the absence of axial backbone the only parameters that can greatly vary as a function of experimental conditions are the length and the relative concentration of the different species present in solution. Indeed, SAXS is particularly appropriate to derive the size and shape of particles in solution, and to assess their structural properties. Moreover, in the presence of an equilibrium between different particles, the concentration of different aggregates can be provided at each experimental condition [6,10,24,25].

As a result, the formation and the elongation of G-quadruplexes resulted to be controlled by GMP content and cation type and concentration. In the case of K${}^{+}$, G-quadruplex melting curves were also obtained by SAXS and validated by FTIR spectroscopy measurements.

## 2. Results

We investigated by SAXS GMP self-assembly occurring in aqueous diluted solutions at different concentrations, and in the presence of different type and amount of monovalent counter ions. The complete list of the investigated samples is shown in Table 1.

**Figure 2:**
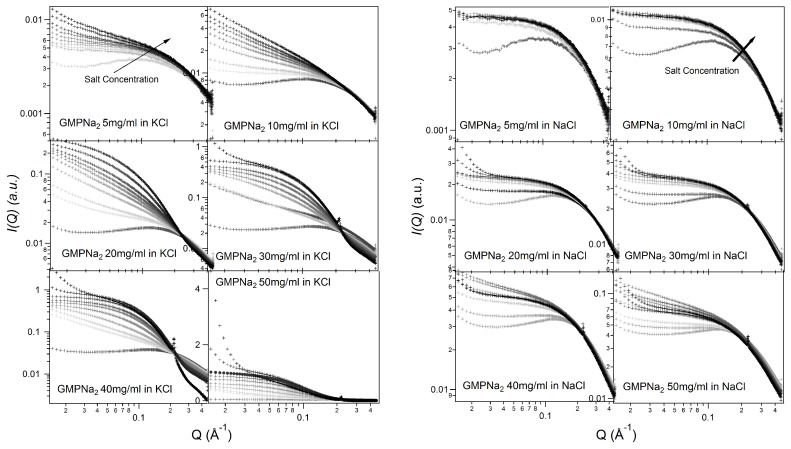
SAXS profiles for GMPNa${}_{2}$ in KCl and NaCl solutions at 24 °C. In each frame, GMP concentration is reported. The grey color of the lines is related to the salt molarity of the solution, that increases, as the arrow indicates, from 0.06 M up to 1.0 M.

**Figure 3:**
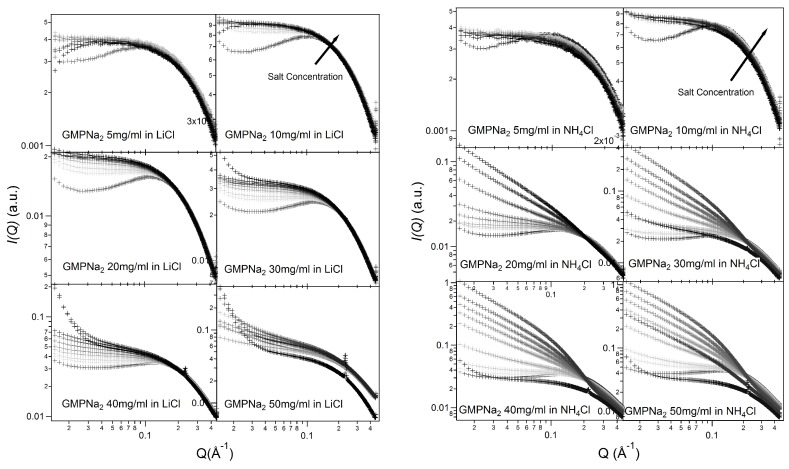
SAXS profiles for GMPNa${}_{2}$ in LiCl and NH${}_{4}$Cl solutions at 24 °C. In each frame, GMP concentration is reported. The grey color of the lines is related to the salt molarity of the solution, that increases, as the arrow indicates, from 0.06 M up to 1.0 M.

**Figure 4:**
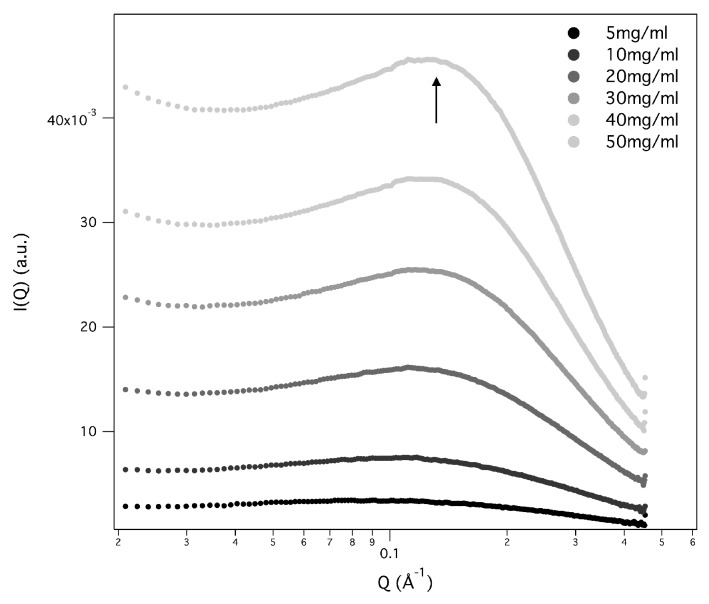
SAXS profiles for GMPNa${}_{2}$ in pure water at 24 °C. The grey color of the lines is related to GMP concentration, as in the legend. The arrow indicates the observed correlation band.

Figure 2 and Figure 3 show the SAXS results obtained at different GMPNa${}_{2}$ concentrations and in the presence of the different salts. Data are grouped by GMP concentration, and clearly show that the overall intensity increases by increasing the ionic strength of the solution and the GMP concentration. Moreover, a Bragg peak appears at the higher investigated concentrations and ionic strengths. Such results suggest that self-assembly proceeds as far as GMP concentration increases, but also that excess counterions play an important role. In pure water (see Figure 4), a large interaction band at Q=0.12 Å${}^{-1}$, whose intensity increases with GMP concentration, is detected. In the absence of an effective screening due to counterions, the high electrostatic repulsion among the negatively charged particles (probably GMP and G-quartets, whose number increases with concentration) occurs and it is substantial. In the presence of K${}^{+}$ ions, the intensity of SAXS curves at low *Q* increases, proving that the aggregation processes have been activated, and G-quadruplexes form. Accordingly, SAXS curves show the typical form expected for cylindrical particles (see below), and, as far as the ionic strength increases, the elongated quadruplexes collapse into stable lyotropic phases, due to a strong reduction of repulsive interactions.

**Figure 5:**
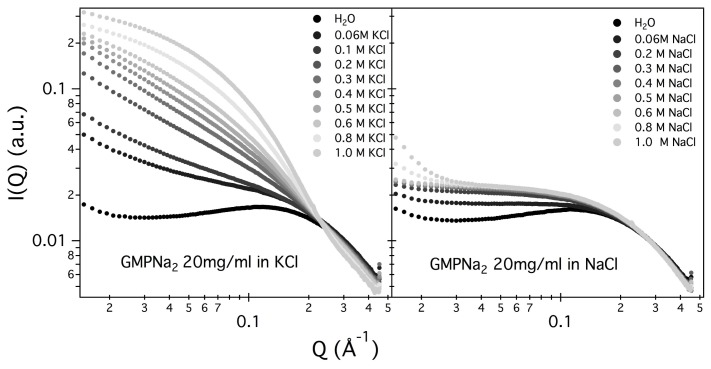
Comparison of SAXS profiles for GMPNa${}_{2}$, 20 mg/mL, in KCl and NaCl solutions.

Results obtained in the presence of sodium ions are rather different, because the increasing of the scattering at low *Q* values is really less evident in respect to the potassium ions case (see Figure 5, which compares at the same scale SAXS data from GMPNa${}_{2}$ samples in KCl and NaCl at the same concentration). Along the entire range of explored GMP concentrations, the presence of Na${}^{+}$ promotes the formation of G-quadruplexes, but further nucleation and elongation processes are probably very scarce.

Similar results have been obtained in LiCl and NH${}_{4}$Cl (see Figure 3). As already reported [20], the size of the cation and its energy of dehydration are the main factors that contribute to cation ability in stabilizing the G-quadruplex structure. As a rule, the dehydration energy of monovalent cations is inversely proportional to their ionic radii (Li${}^{+}$ < Na${}^{+}$ < NH${}_{4}^{+}$ < K${}^{+}$). However, the free energy of dehydration should combine with the free energy of ion coordination within the G-quartets. Hence, the larger ability of potassium to stabilize quadruplexes in respect to Na${}^{+}$ ions is driven firstly by the net difference in favour of K${}^{+}$ between the greater energetic cost of Na${}^{+}$ dehydration with respect to K${}^{+}$ dehydration, and secondly by the intrinsic free energy of ion coordination within G-quartets, which is actually more favourable for Na${}^{+}$ than for K${}^{+}$ [26]. The trend observed in the SAXS curves fully supports these arguments about Na${}^{+}$ *versus* K${}^{+}$ selectivity.

It should also be observed that the negatively charged phosphates, located on G-quadruplexes surface, provide the bulk of metal ion interaction sites which cause non-specific charge neutralization. Indeed, independently of the type of cation, when the salt concentration increases above 0.5 M, the excess of counterions strongly reduces the negative charges, so that G-quadruplexes interact closer. Indeed, the appearing of Bragg peaks indicate the formation of stable lyotropic phases (see last frames of Figure 2 and Figure 3. See also below).

### 2.1. Guinier Analysis

SAXS curves were first analyzed applying the Guinier law:

(1) $$I\left(Q\right)≅I\left(0\right)\exp\left(-\frac{R_{g}^{2}Q^{2}}{3}\right)$$

where I(0) is the scattering intensity at Q=0 and $R_{g}$ is the gyration radius of the scattering particles, which provides an estimation of the overall size of the particles in solution. The validity of the Guinier law is expressed by the linearity in the low-*Q* region of the plot of log[I(Q)] as a function of $Q^{2}$. In general, this approximation is applied up to a maximum value of *Q* according to the condition $R_{g}Q_{\max}\leq 1.3$ [27].

Basically, the Guinier’s law can be correctly applied when the system is constituted by diluted particles with an isotropic shape (globular-like particles). When the system is mainly constituted by elongated, randomly oriented rod-like particles, the Guinier equation becomes:

(2) $$Q\;I\left(Q\right)≅A\;\exp\left(-\frac{R_{c}^{2}Q^{2}}{2}\right),$$

where *A* is a constant and $R_{c}$ is the cross-sectional radius of gyration, which is related the overall size of the cross section of the particle. For an elongated particle of circular section *R* and constant electron density, $R_{c}$ and *R* are related by $R_{c}^{2}=R^{2}/2$. As before, the rod-like Guinier approximation has a limited validity and can be applied in the range of *Q* where the plot of log[QI(Q)] versus $Q^{2}$ shows a linear trend.

**Figure 6:**
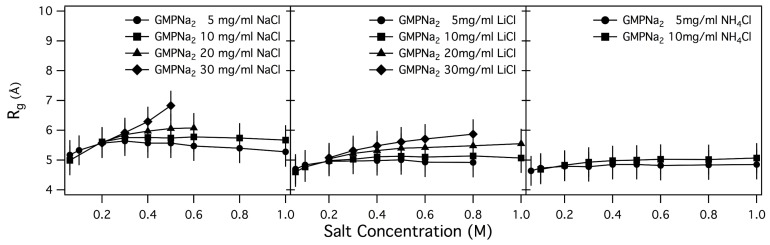
Ionic strength dependence of $R_{g}$ measured from GMPNa${}_{2}$ samples prepared in NaCl, LiCl and NH${}_{4}$Cl. GMPNa${}_{2}$ concentrations are reported in each frame.

**Figure 7:**
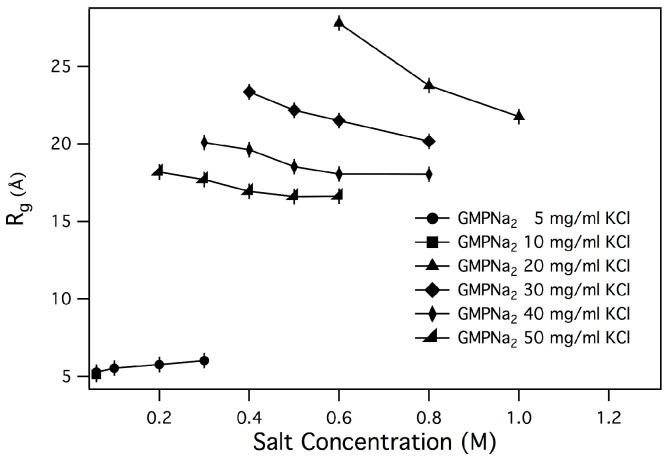
Ionic strength dependence of $R_{g}$ measured from GMPNa${}_{2}$ samples prepared in KCl. GMPNa${}_{2}$ concentrations are reported in the legend.

By performing a linear fit of log[I(Q)] *vs.* $Q^{2}$ data, the $R_{g}$s values reported in Table 2 have been obtained, and plotted in Figure 6 and Figure 7 as function of salt and GMP concentration.

As a general result, it can be observed that only small changes in the radius of gyration are detected by increasing the ionic strength, while an increase of GMP concentration leads to an increase of $R_{g}$. Changes are small in the case of samples prepared in NaCl, LiCl and NH${}_{4}$Cl, while $R_{g}$s for samples prepared in KCl are surprisingly larger, confirming the already suggested capability of K${}^{+}$ to induce aggregation. Even if in the presence of chemical equilibrium between different aggregate species (as in the present case, illustrated in Figure 1) the application of the Guiner law can lead to unphysical averaged parameters, it can be suggested that an increase of the size of the particles in solution or a change in the relative amount of smaller and larger aggregates occurs when the GMPNa${}_{2}$ concentration increases [10,28]. Indeed, the calculated radii of gyration of a GMP molecule and a G-quartet are 5.0 and 9.5 Å, respectively.

By linear fitting log[QI(Q)] *vs.* $Q^{2}$ data, $R_{c}$ values were also obtained. In any case, when the fitting was possible (e.g., GMPNa${}_{2}$ concentrations larger than 10 mg/mL and ionic strength larger than 0.1 M), $R_{c}$ resulted approximately constant and equal to 9.2 Å (with an error of 10%).

From the radii of gyration, the length of G-quadruplexes was estimated. Indeed, by approximating the quadruplex shape to a cylinder, the length of the particle can be derived by:

(3) $$L^{2}=12\;\left(R_{g}^{2}-R_{c}^{2}\right).$$

The length of G-quadruplexes estimated by using Equation (3) for GMPNa${}_{2}$ dissolved in KCl are reported in Table 3. As expected, the length depends on GMP and K${}^{+}$ concentrations, but a clear trend cannot be derived, probably because $R_{g}$s values cannot capture the complex quadruplex formation, elongation and fragmentation processes.

### 2.2. Model Data Analysis

**Figure 8:**
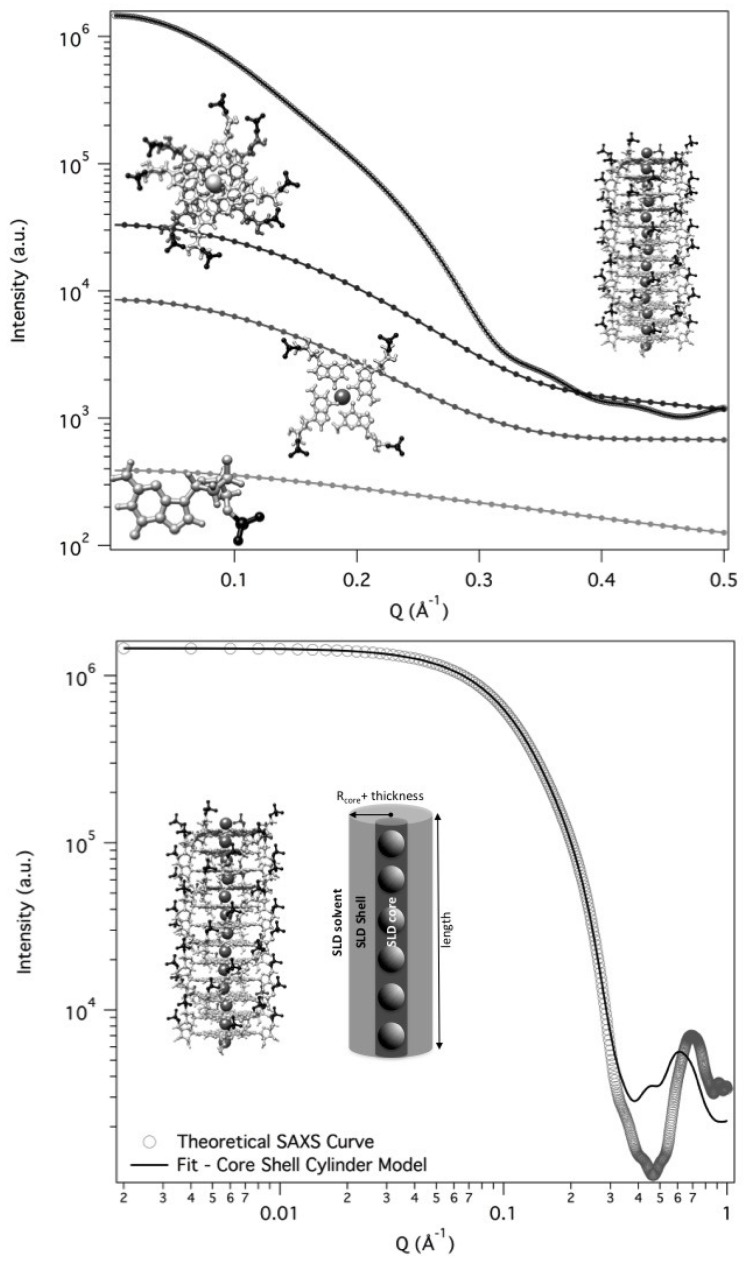
Top frame: theoretical SAXS curves for GMP, G-quartet, G-octamer and a dodecameric G-quadruplex obtained by GENFIT from atomic models. Lower frame: best fit of the theoretical dodecameric G-quadruplex SAXS curve (open circles) with a core-shell cylinder model (continuous line). Note that the fitting fails just at *Q* values higher than 0.4 Å${}^{-1}$, where the ratio between signal and noise of an in-solution SAXS curve is usually low. Fitting parameters were: $R_{\mathrm{core}}=2.0\pm 0.3$ Å, δ=11.4±0.4 Å, L=41.9±0.5 Å, $\rho_{\mathrm{core}}=\left(0.56\pm 0.02\right)$ e/Å${}^{3}$, $\rho_{\mathrm{shell}}=\left(0.47\pm 0.01\right)$ e/Å${}^{3}$.

Since the complex self-assembling mechanism and because the formed G-quadruplexes could be polydisperse in length, any simple method selected to fit the scattering curves needs to be validated. In the present case, the G-quadruplex can be best represented by using a core-shell cylinder model: the particle is considered to be a right circular cylinder, with a length *L* and a core-shell electron density profile. The core, of radius $R_{\mathrm{core}}$ and electron density $\rho_{\mathrm{core}}$, and the shell, of thickness δ and electron density $\rho_{\mathrm{shell}}$, are considered to be uniform over the entire cross section of the cylinder. The core-shell cylinder model was validated considering theoretical SAXS profiles for randomly oriented GMP and G-particles. The calculation was performed by using the GENFIT software [29] and atomic models for the G-quartet, the G-octamer and a G-quadruplex made by 12 stacked G-quartets generated by simple modeling packages from the PDB crystallographic structure of DNA quadruplex GCGGTGGAT (PDB id 1NYD [30]). According to the SASMOL method exploited in the GENFIT software [31], the mass density of the hydration water was considered 5% greater than the one of bulk water. Results are shown in Figure 8 (top frame).

A Guinier analysis over the theoretical SAXS curves was then performed to check if the corresponding radii of gyrations agree with the model data. In particular, for the dodecameric G-quadruplex the measured $R_{g}$ and $R_{c}$ were used to recover the particle length (and the number of G-quartet stacked): being $R_{g}=15.4$ Å and $R_{c}=9.2$ Å, *L* resulted 44.5 Å, which corresponds to a number of stacked G-quartet equal to L/3.4 Å = 12.6 (3.4 Å is the stacking distance, as determined in liquid crystalline phases [32]).

To validate the core-shell cylinder model, we then fitted the SAXS theoretical curve for the dodecameric G-quadruplex. The best fitting curve is reported in Figure 8 (lower frame): best fitting parameters indicated a cylinder length of 41.9±0.5 Å, which is fully compatible with the staking of 12 G-quartet at the repeat distance of 3.4 Å. Moreover, all the other fitting parameters (in particular, inner radius of 2.0 Å, shell thickness of 11.4 Å and electron densities of the different regions) were fully compatible with the G-quadruplex model.

We then used the core-shell cylinder model to analise the whole set of SAXS curves, with the aim to associate G-quadruplexes lengths to the sample composition, in terms of kind and amount of ions. Since all the GMPNa${}_{2}$ investigated samples contain quadruplexes of different length but similar cross-section, all the set of SAXS curves have been simultaneously analyzed by means of the global fitting GENFIT option [29], considering as common parameters the core radius $R_{\mathrm{core}}$, the shell thickness δ, the corresponding electron densities ($\rho_{\mathrm{core}}$ and $\rho_{\mathrm{shell}}$), as well as a unique calibration factor.

**Figure 9:**
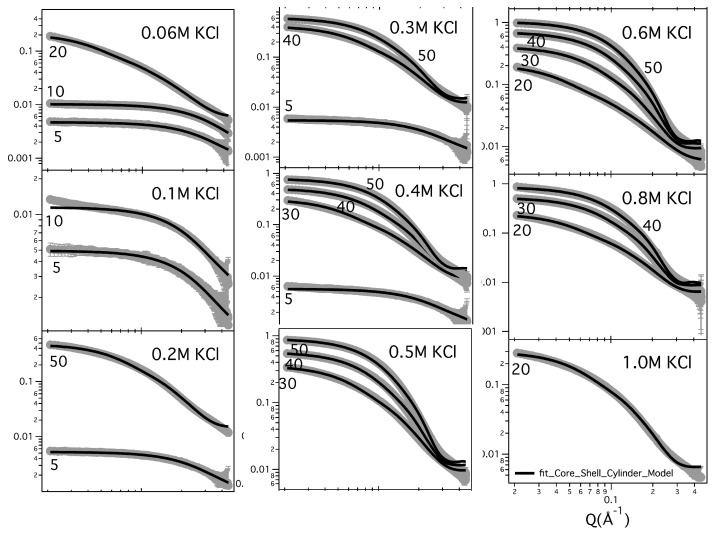
Experimental and model fitting SAXS curves for GMPNa${}_{2}$ samples prepared in KCl solution. Common fitting parameters were: $R_{\mathrm{core}}=1.8\pm 0.2$ Å, δ=11.2±0.4 Å, $\rho_{\mathrm{core}}=\left(0.57\pm 0.01\right)$ e/Å${}^{3}$ and $\rho_{\mathrm{shell}}$ = (0.46 ± 0.02) e/Å${}^{3}$. In each frame, GMPNa${}_{2}$ (in mg/mL) and KCl (in M) concentrations are indicated.

**Figure 10:**
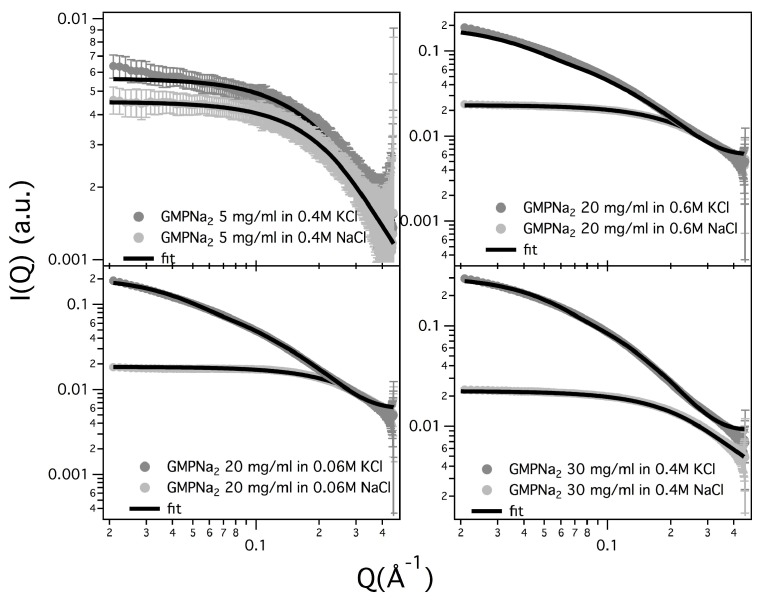
Comparison of experimental and model fitting SAXS curves for a few GMPNa${}_{2}$ samples prepared in KCl and in NaCl solutions. Common fitting parameters were $R_{\mathrm{core}}=1.8\pm 0.2$ Å, δ=11.2±0.4 Å, $\rho_{\mathrm{core}}=\left(0.57\pm 0.01\right)$ e/Å${}^{3}$ for K${}^{+}$, $\rho_{\mathrm{core}}=\left(0.32\pm 0.01\right)$ e/Å${}^{3}$ for Na${}^{+}$ and $\rho_{\mathrm{shell}}=\left(0.46\pm 0.02\right)$ e/Å${}^{3}$.

As an example, the fitted curves related to GMPNa${}_{2}$ in KCl samples are reported in Figure 9, while a few comparisons for samples prepared in KCl and in NaCl are shown in Figure 10. In any case, the quality of the fit can be appreciated, confirming that all the curves can be very well reproduced by considering the core-shell cylinder model. The corresponding G-quadruplex lengths are reported in Table 4.

Fitted lengths evidence how the G-quadruplex length in the presence of Na${}^{+}$ remains almost constant (about 6 stacked G-quartets), even varying the GMP or the NaCl concentrations, in agreement with the previous Guinier analysis. The situation is similar in the presence of Li${}^{+}$ or NH${}_{4}^{+}$, even if quadruplexes result slightly shorter (made by 4 to 5 G-quartets). By contrast, G-quadruplexes appear made by 9 to 30 stacked G-quartets in the presence of KCl, but surprisingly the number of stacked G-quartets decreases by increasing both GMP and KCl concentrations. Such a finding seems to indicate that at constant counterion concentration, the addition of GMP determines the formation of a larger number of short cylinders (nucleation), while elongation is hindered, probably because in this condition the whole system has a greater thermodynamic stability.

### 2.3. Condensed Phases

**Figure 11:**
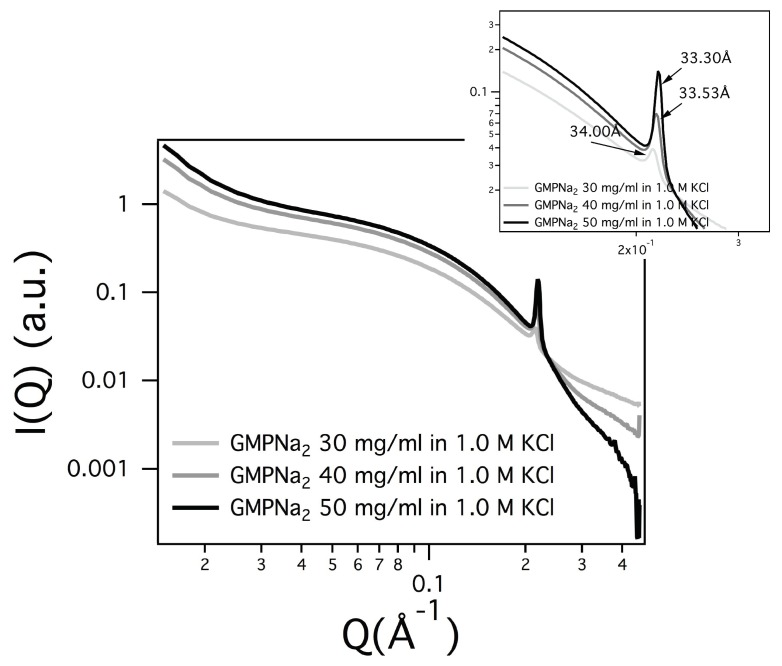
SAXS curves for GMPNa${}_{2}$ samples prepared at 30, 40 and 50 mg/mL in 1 M KCl. The inset focuses on the Bragg peaks: the corresponding hexagonal unit cell dimensions are indicated.

In several SAXS curves, a Bragg peak appears (see Figure 11), suggesting that the particle number density is so high, and the particle-particle repulsive interactions so low, that the G-quadruplexes adopt a liquid-crystalline order [7,10,32]). Because in all the different cases only one Bragg peak was detected, peak indexing was not possible, but previous structural results [32] provided continuity arguments to describe the liquid-crystalline phase. The observed Bragg peak was then considered the first order of a 2D hexagonal pattern, so that the unit cell dimension (which in the hexagonal phase corresponds to the lateral distance between two parallel G-quadruplexes) was derived. As expected, the unit cell parameter depends on concentration (the unit cell decreases when the GMPNa${}_{2}$ increases, see the parameters reported in Figure 11). Moreover, because the screening effect due to counterions, the unit cell dimension decreases when the salt concentration increases (e.g., for GMPNa${}_{2}$ 50 mg/mL sample, the unit cell reduces from 33.8 to 33.5 Å when KCl content changes from 0.8 to 1 M; in the same condition, the unit cell of the GMPNa${}_{2}$ 40 mg/mL sample shows a decrease from 33.5 to 33.3 Å).

### 2.4. Temperature Effect

**Figure 12:**
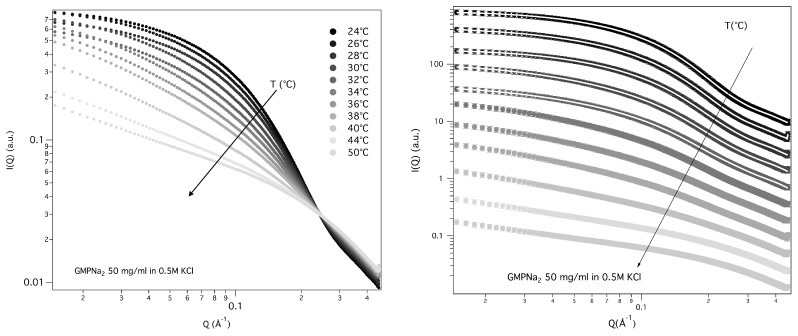
Temperature dependence of the SAXS curves for GMPNa${}_{2}$ 50 mg/mL in 0.5 M KCl. In the right panel, the curves are scaled for clarity, as the model fitting to the curves measured from 24 to 32 °C is shown.

The structural properties of GMPNa${}_{2}$ 50 mg/mL prepared in 0.5 M KCl were also investigated as a function of temperature, from 24 to 50 °C. SAXS data, reported in Figure 12, show that the overall SAXS intensity decreases on heating, suggesting that high-temperatures destabilise the aggregates and determine quadruplex fragmentation.

Guinier’s approximation was used to derive the corresponding particle $R_{g}$s, while the quadruplex length was estimated both by using gyration radius relationships (see Equation (3)) and by the core-shell cylinder model fitting. All results are reported in Table 5. Note that a fitting procedure has been provided only for the first five curves, because when a significant fragmentation of the G-quadruplexes occurs, sample polydispersion and low scattering intensity does not result in a good fit.

**Figure 13:**
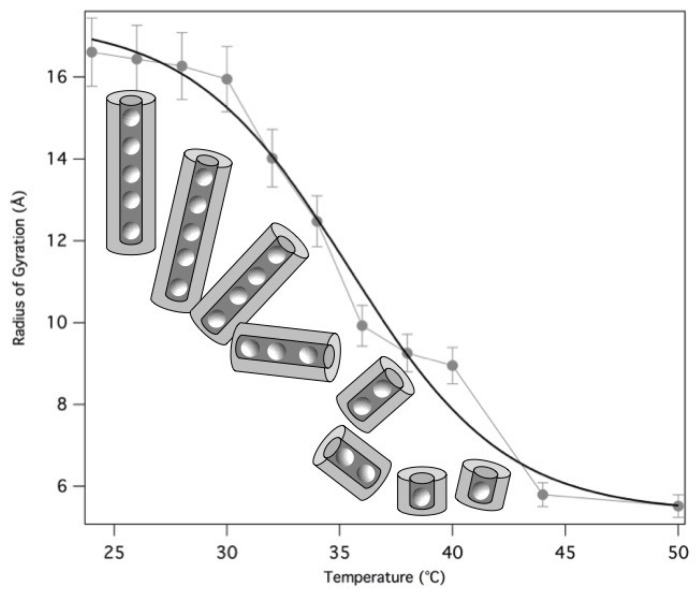
Temperature dependence of the radius of gyration measured in GMPNa${}_{2}$ samples prepared at 50 mg/mL in 0.5 M KCl. The quadruplex breaking as a function of temperature is illustrated. The continuous line models the fragmentation process by a Hill equation [33].

The derived radii of gyration are reported as a function of temperature in Figure 13. The fragmentation can be modelled by a Hill equation [33], suggesting that the binding of G-quartets on the quadruplexes is cooperative.

A further thermal analysis has been performed in the special case of GMPNa${}_{2}$ prepared at 80 mg/mL in KCl solutions. In this case, SAXS results, in terms of $R_{g}$s measured as a function of temperature at different KCl molarities, have been compared to FTIR spectroscopy results obtained in the same conditions and based on the evaluation of the frequency changes of the C${}_{6}$=O${}_{6}$ carbonyl stretching modes of the guanine [34]. Indeed, it has been demonstrated that this band is sensitive to the guanine structuring, since it moves from about 1693 cm${}^{-1}$, found in DNA quadruplex, to 1655–1670 cm${}^{-1}$, observed for single stranded DNA [35,36]. Similar results were also observed in the case of GMPNa${}_{2}$ at basic pH: the self-association in quadruplexes was observed to determine a blue shift from 1657 cm${}^{-1}$ to ≃1675 cm${}^{-1}$[37]. Therefore, the temperature dependence of the frequency of the C${}_{6}$=O${}_{6}$ stretching modes could be a good indication for the assembling/disassembling of GMP in solution.

**Figure 14:**
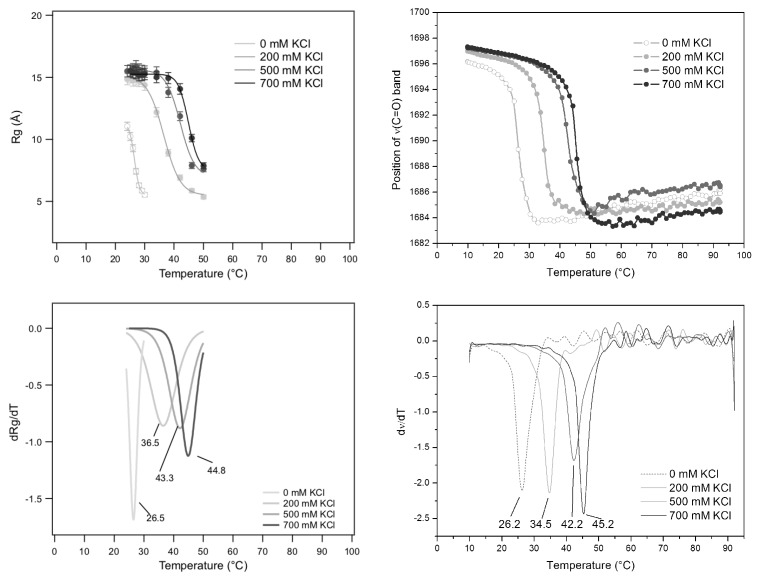
Comparison between SAXS and FTIR results.

FTIR and SAXS results are reported in Figure 14. First, the temperature-induced behaviour compares well with the one already described in Figure 13 for GMPNa${}_{2}$ (50 mg/mL and 0.5 M KCl), the main difference being the inflection-point temperature ($T_{m}$): the more the GMP concentration increases, the more the inflection-point temperature rises. Second, at the considered GMP concentration of 80 mg/mL, both $R_{g}$s and carbonyl stretching modes of guanine show a very similar temperature dependence, indicating that in both cases the quadruplex fragmentation process is monitored. However, the temperature at the flex shows a strong dependence on KCl molarity, suggesting that the cation concentration controls the G-quadruplex disassembling temperature. A negative exponential function fitted to the melting temperatures as a function of KCl concentration led to $T_{m,KCl}=(48.3\pm 1.5)$ °C, $T_{m,wat}=(26.3\pm 2.3)$ °C and τ=(0.370±0.056) M ($\chi^{2}$ = 2.75), which indicate a strong effect on G-quadruplex thermal stability ($T_{m}$ increases more than 22 °C, passing from 0 to 1 M KCl). Considering a very elementary model for the *diffusively bound* layer of ions around G-quadruplexes [38], it can be calculated that the exponential constant τ corresponds to the molar concentration of G-quartets in solution (0.055 M) added to the molar concentration of the 75% of GMP charges (0.33 M). Because osmotic stress experiments performed on the same system indicated that up to 1 M KCl, there are 12% of G-quadruplex charges not balanced by counter-charges [39,40], the strong sensitivity observed here for K${}^{+}$ is probably determined not only by its preferred binding to the inner G-quartet sites, but also by a high degree of very effective and specific charge neutralization on the G-quadruplex surface.

## 3. Discussion and Conclusions

Supramolecular self-assembly has revealed itself over the time as a singular mechanism to devise complex architectures built through non-covalent forces. On this subject, many efforts have been devoted to exploit H-bonds as a key for encoding chemical information, and the structures expressed have spanned the range of dimensions and shapes. Guanosines and guanosine derivatives were shown to undergo a hierarchical self-assembly process in water and in organic solvents [6,7]. The process is essentially driven by ion complexation and solvophobic effects, resulting in nanoribbons [6,7] or in a stack of disks, which define the helicoidal nanowire (G-quadruplex) of 25 Å diameter and up to hundred nanometers long with an open central channel [10]. Noteworthy, guanosine-rich single-stranded sequences of DNA have been demonstrated to adopt various tertiary structures, including G-quadruplexes.

The biological role of such sequences and the structural properties of G-quadruplexes have been extensively discussed, but the understanding of basic physical properties is still limited, in particular for which concern the principles that govern quadruplex formation and stability, and counterion effects.

As the problem is really complex, the G-quadruplex formation pathway and the G-quadruplex structural properties in solution and in condensed phases have been studied using a simple model obtained by self-assembling of a free single molecules of GMP: in this case, the axial sugar-phosphate backbone is absent and any effect relating thermal and thermodynamical stability to counterions, hydrational properties and hydrophobicity, phase behaviour and so on should be necessarily amplified. Therefore, the extended study reported here can probably help for a better understanding of the self-assembly process, identifying the key parameters involved. Several types of counterions and counterion combinations as well as a wide range of concentrations, temperatures, ionic strengths and even solvent characteristics were explored. The nucleation and elongation properties of G-quadruplexes formed using GMP sodium salt in the presence of different excess counterions have been analyzed by using SAXS technique. Experiments have been performed as a function of temperature and sample composition and concentration, and the effects of one parameter at a time have been considered.

**Figure 15:**
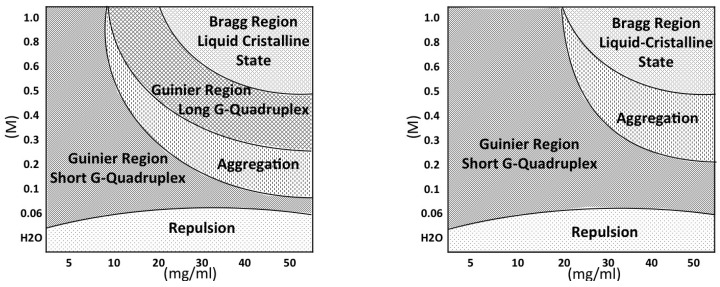
Phase diagrams for GMPNa${}_{2}$ in K${}^{+}$ (left side) and in Na${}^{+}$ (right side). *Repulsion*: very short, strongly interacting quadruplexes. A correlation band characterise the SAXS curves; *Guinier Region—Short G-Quadruplex*: short quadruplexes. SAXS curves show an extended Guinier region; *Aggregation*: elongated, polydisperse quadruplexes. In the SAXS curves, the Guinier region is lost; *Guinier Region—Long G-Quadruplex*: elongated, monodisperse quadruplexes. SAXS curves show an extended Guinier region; *Bragg Region—Liquid-Crystalline State*: long quadruplexes, packed according a partially ordered hexagonal lattice. SAXS curves show Bragg peaks.

The first, expected result is the evidence that the G-quadruplex formation is controlled by a delicate equilibrium between GMP concentration and counterion content. Both GMP concentration and excess cation content favour the formation and the elongation of G-quadruplexes, but the two processes are differently driven by the two factors and the final effect also combines with the counterion selectivity. The main effects are better represented by the phase diagrams shown in Figure 15. In the presence of K${}^{+}$ (left diagram), there is a first region in which relatively short quadruplexes form. Particle repulsion is strong and a correlation band is observed on SAXS data. Salt addition appears to favour either the G-quadruplex elongation and the nucleation process. At the same time, the repulsive forces reduce. Accordingly, SAXS curves show an extended Guinier region. By increasing GMP concentration, the elongation proceeds to the formation of quadruplexes with a very polydisperse length: accordingly, the Guinier regime is lost. By further increasing the GMP concentration and/or the ionic strength, a third region in which cylinders appear considerably long (about 30 to 100 Å) and rather uniform, so that the Guinier regime is reactivated, is reached. In this condition, elongation and fragmentation of quadruplexes probably compensate. Finally, in the right, top side of the phase diagram (high GMP composition, high ionic strength) the formation of the lyotropic liquid crystalline phase is indicated. Because the crowding condition and the high ionic screening effect, G-quadruplexes collapse into a 2D hexagonal structure. In the case of Na${}^{+}$ (but this is also valid for Li${}^{+}$ and NH${}_{4}^{+}$), the phase diagram is modified. The first region (*repulsion*, in which strongly interacting short quadruplexes occur) is very similar to that of K${}^{+}$. By contrast, the Guinier region, where short G-quadruplexes exist and in which nucleation and not elongation mainly occur both as a function of GMP concentration and cation content, extends in a broad portion of the phase diagram. Indeed, because G-quadruplex elongation is limited, the aggregational region is relatively small and is observed only at large GMP compositions and ionic strengths, immediately followed by the liquid-crystalline ordered hexagonal phase.

In conclusion, the observed behaviour is general: in the absence of external cations, only the presence of GMP monomers is detected, probably because counterions derived from dissociation of GMP salt are not sufficient for triggering aggregation. When counterions are added, self-assembly occurs: indeed, the formation of G-quadruplexes through nucleation and elongation processes is demonstrated considering both the size of radii of gyration and the length of the quadruplexes directly obtained from the fitting procedures. However, it appears that three of the analysed counterions (NH${}_{4}$${}^{+}$, Na${}^{+}$ and Li${}^{+}$) produce a similar effect on the system, i.e., the formation of copious, but short cylinders. A different behaviour is observed when K${}^{+}$ is considered: potassium strongly accelerates the elongation process, leading to long cylinders. In all cases, the occurrence of condensed liquid crystalline phases was detected (as evidenced by the presence of Bragg peaks): the unit cell variations suggested a ‘classical’ behavior: excess counterions switch off (partially) the charges on the external G-quadruplex surface, determining the cylinder approaching and the consequent decrease of the unit cell size when the ionic strength increases. Obviously, this effect is independent of the kind of counterion used.

As a last comment, it should be observed that none of the investigated samples showed turbidity: hence, the possible occurrence of liquid-liquid phase separation was not taken into consideration [41]. However, because recent studies evidenced that the dynamic clustering of cellular factors, enzymes and nucleic acids mediated by liquid-liquid phase separation is related to almost all aspects of cellular functions [42,43], further investigations on detectable turbidity in more concentrated samples or in samples prepared in the presence of other cations (e.g., bivalent) seem really promising.

## 4. Materials and Methods

### 4.1. Sample Preparation

GMP-free acid (MP Biomedicals) was diluted with bidistilled water and the resulting mixture was heated to promote the solubility of the acid. GMPNa${}_{2}$ salts were obtained by acid-base titration with 1 M sodium hydroxide monitoring the pH until until a steady value 9 is reached. Ethanol was then added to induce GMPNa${}_{2}$ precipitation. The solution was centrifuged at 8000 rpm and the supernatant removed. The pellet was washed twice with ethanol and centrifuged again at the same rotation rate, and subsequently lyophilized using ThermoSavant SPD 111V SpeedVac, obtaining a lyophilized powder. Samples for SAXS experiments were prepared dissolving the lyophilized GMPNa${}_{2}$ powder at concentration $c_{\mathrm{GMP}}$ 5, 10, 20, 30, 40 and 50 mg/mL in bidistilled water or in solutions of chloride counterion (NaCl, KCl, NH${}_{4}$Cl and LiCl) at concentrations ranging from 0.06 to 1 M (Table 1) [10].

### 4.2. SAXS Experiments

SAXS experiments were performed at the ESRF in Grenoble (France) using the BM29 beamline. The wavelength λ of the incident beam was 0.99 Å and the investigated range of the modulus of the scattering vector *Q* (Q=4πsinθ/λ, where 2θ is the scattering angle) was comprised between 0.015 and 0.33 Å${}^{-1}$. The samples were loaded in quartz capillaries of 1.8 mm. The explored temperature range was from 24 °C to 50 °C. The scattering intensity was recorded with the Pilatus 1M Detector (ESRF), radially averaged and corrected for dark current, detector efficiency, buffer contribution and sample transmission [44,45,46].

### 4.3. FTIR Experiments

FTIR experiments were performed by using a Bruker IFS-48 FTIR spectrometer. All spectra were taken under air as a function of temperature with 50 scans at a resolution of 0.5 cm${}^{-1}$ and a spectral range of 800–4000 cm${}^{-1}$. Samples were injected into a thermostated cell (GS 20500 cell by Graseby-Specac Ltd, Orpington, Kent, UK) fitted with CaF${}_{2}$ windows separated by a mylar spacer (thickness of 25 μm). To obtain spectra at different temperatures, an external bath circulator (HAAKE F3) was used. The temperature was checked by a thermocouple placed directly onto the windows. Samples were equilibrated for 5 min at each temperature be fore spectra were collected.

## Figures and Tables

**Table 1:** Complete list of investigated samples.

	$c_{\mathrm{GMP}}$ (mg/mL)	Counterion	C (M)	Temperature (°C)
GMPNa${}_{2}$	5–50	-	-	24
GMPNa${}_{2}$	5–50	Na${}^{+}$	0.06–1	24
GMPNa${}_{2}$	5–50	K${}^{+}$	0.06–1	24–50
GMPNa${}_{2}$	5–50	NH${}_{4}^{+}$	0.06–1	24
GMPNa${}_{2}$	5–50	Li${}^{+}$	0.06–1	24

**Table 2:** Radii of gyration (in Å) measured from GMPNa${}_{2}$ samples prepared in NaCl, KCl, LiCl and NH${}_{4}$Cl. Errors are of the order of 10%. The *dash* indicates SAXS profiles to which the Guinier approximation was not applicable, while the words *Bragg Peak* were used to indicate SAXS profiles related to a lyotropic phase.

GMPNa${}_{2}$ (mg/mL)	0.1M	0.2M	0.3M	0.4M	0.5M	0.6M	0.8M	1.0M
**NaCl**								
5	6.0	5.6	5.8	5.5	5.8	5.5	5.5	5.8
10	-	5.7	6.0	6.0	5.9	6.0	5.9	5.4
20	-	5.6	6.1	6.3	6.3	6.4	6.3	Bragg Peak
30	-	5.4	6.1	6.8	7.0	Bragg Peak	Bragg Peak	Bragg Peak
40	-	5.6	6.8	7.6	8.0	Bragg Peak	Bragg Peak	Bragg Peak
50	-	6.8	8.1	8.8	Bragg Peak	Bragg Peak	Bragg Peak	Bragg Peak
**KCl**								
5	5.5	5.8	6.0	-	-	-	-	-
10	-	-	-	-	-	-	-	-
20	-	-	-	-	-	27.8	23.8	21.7
30	-	-	-	23.4	22.2	21.5	20.2	Bragg Peak
40	-	-	20.1	19.6	18.5	18.1	18.1	Bragg Peak
50	-	18.2	17.7	16.9	16.6	16.6	Bragg Peak	Bragg Peak
**LiCl**								
5	4.8	4.9	5.0	5.0	4.9	4.9	5.1	-
10	4.6	4.7	5.0	5.0	5.1	5.1	5.1	5.1
20	-	5.0	5.2	5.3	5.4	5.4	5.5	5.55
30	-	5.1	5.3	5.5	5.6	5.7	5.9	Bragg Peak
40	-	-	-	-	-	-	Bragg Peak	Bragg Peak
50	-	-	-	-	-	Bragg Peak	Bragg Peak	Bragg Peak
**NH_4_Cl**								
5	4.7	4.8	4.7	4.8	4.8	4.8	4.8	4.8
10	4.7	4.8	4.9	5.0	5.0	5.1	5.0	5.1
20	-	-	-	-	-	-	-	-
30	-	-	-	-	-	-	-	Bragg Peak
40	-	-	-	-	-	-	-	Bragg Peak
50	-	-	-	-	-	-	Bragg Peak	Bragg Peak

**Table 3:** Estimation of the length *L* (in Å) of G-quadruplexes occurring in GMPNa${}_{2}$ samples prepared in KCl. Errors are of the order of 5%. Symbols as in Table 2.

GMPNa${}_{2}$	KCl (M)							
(mg/mL)	0.1	0.2	0.3	0.4	0.5	0.6	0.8	1.0
5	−	−	−	−	−	−	−	−
10	−	−	−	−	−	−	−	−
20	−	−	−	−	−	90.9	76.0	68.2
30	−	−	−	74.4	69.9	67.4	62.2	Bragg Peak
40	−	−	61.9	60.1	55.8	53.9	53.8	Bragg Peak
50	−	54.4	52.3	49.3	47.9	48.1	Bragg Peak	Bragg Peak

**Table 4:** G-quadruplex lengths (in Å) obtained by fitting the SAXS data with a core-shell cylinder model. Errors are in the order of 5%. Common fitting parameters were $R_{\mathrm{core}}=1.8\pm 0.2$ Å, δ=11.2±0.4 Å, $\rho_{\mathrm{core}}=\left(0.57\pm 0.01\right)$ e/Å${}^{3}$ for K${}^{+}$, $\rho_{\mathrm{core}}=\left(0.32\pm 0.01\right)$ e/Å${}^{3}$ for Na${}^{+}$, $\rho_{\mathrm{core}}=\left(0.09\pm 0.01\right)$ e/Å${}^{3}$ for Li${}^{+}$, $\rho_{\mathrm{core}}=\left(0.31\pm 0.01\right)$ e/Å${}^{3}$ for NH${}_{4}^{+}$ and $\rho_{\mathrm{shell}}=\left(0.46\pm 0.02\right)$ e/Å${}^{3}$. Symbols as in Table 2.

GMPNa${}_{2}$ (mg/mL)	0.06M	0.1M	0.2M	0.3M	0.4M	0.5M	0.6M	0.8M	1.0M
**NaCl**									
5	17.9	18.4	19.3	19.3	19.1	19.0	18.9	18.4	18.4
10	16.5	-	19.2	19.5	19.5	19.6	19.6	19.4	19.2
20	14.9	-	18.7	19.6	19.9	20.2	20.3	-	Bragg Peak
30	-	-	18.3	19.6	20.7	-	Bragg Peak	Bragg Peak	Bragg Peak
40	-	-	-	-	-	-	Bragg Peak	Bragg Peak	Bragg Peak
50	-	-	-	-	-	Bragg Peak	Bragg Peak	Bragg Peak	Bragg Peak
**KCl**									
5	18.8	19.5	20.2	21.2	22.5	-	-	-	-
10	19.4	21.2	-	-	-	-	-	-	-
20	21.7	-	-	-	-	-	102.5	81.7	66.7
30	-	-	-	-	79.8	63.7	53.7	44.9	Bragg Peak
40	-	-	-	63.1	47.2	41.5	36.8	36.7	Bragg Peak
50	-	-	60.5	40.1	32.6	30.8	30.1	Bragg Peak	Bragg Peak
**LiCl**									
5	15.5	16.2	16.8	-	17.0	17.0	-	-	-
10	14.5	15.5	16.7	17.0	17.3	17.5	-	-	-
20	13.2	14.7	16.5	17.4	17.9	18.2	-	-
30	-	-	16.4	-	18.2	18.7	-	-	Bragg Peak
40	-	-	-	-	-	-	-	Bragg Peak	Bragg Peak
50	-	-	-	-	-	-	Bragg Peak	Bragg Peak	Bragg Peak
**NH_4_Cl**									
5	15.1	15.7	16.2	16.1	16.4	16.3	16.2	16.5	16.5
10	14.2	15.2	16.0	16.7	16.9	17.1	17.2	17.3	17.4
20	13.2	14.5	16.2	18.8	-	-	-	-	-
30	-	-	-	-	-	-	-	-	Bragg Peak
40	-	-	-	-	-	-	-	-	Bragg Peak
50	-	-	-	-	-	-	-	Bragg Peak	Bragg Peak

**Table 5:** Temperature dependence of $R_{g}$s and particle length for GMPNa${}_{2}$ 50 mg/mL in 0.5 M KCl. Errors are in the order of 5%.

Temperature	$R_{g}$	Length from $R_{g}$ and $R_{c}$	Length from Model Fitting
°C	(Å)	(Å)	(Å)
24	16.6	47.9	51.5
26	16.4	47.1	50.3
28	16.3	46.6	49.7
30	15.9	44.9	46.1
32	14.0	36.6	43.2
34	13.5	34.2	-
36	9.5	8.2	-
38	9.2	-	-
40	9.3	-	-
44	5.8	-	-
50	5.5	-	-
